# Modulation and distribution of extracellular free water and tract deficits in rhesus macaques before and after the initiation of emtricitabine + tenofovir disoproxil fumarate + dotutegravir treatment

**DOI:** 10.3389/fimmu.2025.1463434

**Published:** 2025-02-28

**Authors:** Benedictor Alexander Nguchu, Yu Lu, Yifei Han, Yanming Wang, Jiaojiao Liu, Hongjun Li, Peter Shaw

**Affiliations:** ^1^ Oujiang Laboratory (Zhejiang Lab for Regenerative Medicine, Vision, & Brain Health), Wenzhou Medical University, Wenzhou, Zhejiang, China; ^2^ School of Ophthalmology and Optometry and Eye Hospital, Wenzhou Medical University, Wenzhou, China; ^3^ Center for Biomedical Imaging, University of Science and Technology of China, Hefei, Anhui, China; ^4^ Department of Radiology, Beijing Youan Hospital, Capital Medical University, Beijing, China

**Keywords:** 7-stage SIV follow-up study, Chinese-origin rhesus monkeys, simian immunodeficiency virus, EMTBL/FTC + DTG + TDF regimen, viral rebound, drug resistance, extracellular free water volume fraction, diffusivity properties of fiber tissues

## Abstract

**Introduction:**

Understanding the specific timing of cART initiation, its effectiveness, and failures, as well as assessing how well the current cART regimens control viral replication and rebound, enhance immune function, and repair or curb early injury in the central nervous system (*CNS*), is crucial to improving the livelihood of people living with HIV.

**Methods:**

Here, we use an animal model to provide controlled environments to understand how the bodies of Chinese-origin rhesus monkeys, both the immune system and *CNS*, respond to a combination of emtricitabine (*EMTBL/FTC*), dolutegravir (*DTG*), and tenofovir disoproxil fumarate (*TDF*) following the induction of Simian Immunodeficiency Virus (*SIV*). We injected the rhesus monkeys with a dose of *SIV*mac239 (i.e., TCID50—a 50-fold half-tissue culture infective dose) through brachial veins and conducted seven follow-ups at baseline, day 10, day 35, day 84, day 168, day 252, and day 336 for MRI imaging and blood/CSF assays of *SIV* copies and immunity levels.

**Results and discussion:**

Our experimental data demonstrate that the immune system is compromised as early as 7 days after infection, with a rapid rise of *SIV* copies in *ml* and a significant drop of *CD4/CD8* ratio below ~1 within the first 14 days of infection. The alterations in the extracellular environments, manifesting as increased free water volume fraction (*FW-VF*) in MRI data and changes in the diffusivity properties of fiber tissues appearing in *FW*-corrected *FA* and *FW*-corrected *MD*, occur in parallel with an compromised immune system, suggesting that *SIV* enters the brain parenchyma in the early days of infection via a weakened brain defense system, causing inflammatory processes affecting the *CNS*. Our findings demonstrate that our current FTC+*TDF*+*DTG* regimen can enhance the immune system, suppress *SIV* replication, and slow damage to the intra- and extracellular environments. However, it is still ineffective in controlling viral rebound and experiences resistance in some rhesus monkeys, which may lead to further damage to the *CNS*. Our findings also provide the first SIVmac239-based evidence that extracellular *FW-VF* may be a more reliable biomarker of abnormal inflammatory processes, thus providing a better understanding of *SIV* disease progression than previously anticipated.

## 1 Introduction

Experimental limitations posed by human experiments can be easily addressed through controlled environments of animal models. Evidence shows that key pathological mechanisms observed in humans— such as viral replication, immune response, and neuroinflamation— can be replicated in animal models (1–4). Here, we inject rhesus monkeys with simian immunodeficiency virus (*SIV*) to investigate how *SIV* infection influences the distribution of extracellular (ex) free water (exFW) along the major fiber pathways during the initial and later stages of infection. The insights obtained from this investigation are crucial for understanding the ramifications of human deficiency virus (HIV), which is well studied through *SIV* in animal models. We further examine whether changes in the distribution of exFW can be reversed or exacerbated further when macaques are subjected to a treatment regimen. Through this, we can gain a deeper understanding of how drugs impact biological reactions and uncover the potential drawbacks linked to the current therapeutic regimens, the information which is pivotal in refining the medication strategies for better outcomes.

To this end, we specifically designed two sets of rhesus monkeys, each group consisting of five members (N=5/group), and exposed them to distinct conditions. For the first group, we injected the rhesus monkeys with only a SIVmac239 dose and monitored its progression over time. This group serves as the control group, allowing us to study potential variations in the brain’s extracellular milieu after *SIV* infection. For the second group, after injecting the rhesus monkeys with a dose of SIVmac239, we administered a dose of emtricitabine (*EMTBL/FTC)* + tenofovir disoproxil fumarate (*TDF*) + dotutegravir (*DTG)* 40 days after *SIV* infection. Using this group, we observe the potential effects of this dosage in rectifying the detrimental impact of *SIV* on the extracellular environment and preventing further damage to the brain. In particular, we carry out 7-stage follow-up study design to investigate five distinct biological aspects in a cohort of 10 rhesus monkeys. First, we seek to determine the SIV entry into the CNS compartment as early as 7 days post-inoculation. Second, we investigate when the effects of *SIV* begin to manifest in extracellular environment and test whether or not the effects occur in parallel to immune deterioration. Third, we investigate the extent of injury to which the extracellular environment could sustain over extended period of time when the rhesus monkeys are not administered to ant-*SIV* regimen. Fourth, we test whether or not the combination of emtricitabine (*EMTBL/FTC*) + dotutegravir (*DTG*) + tenofovir disoproxil fumarate (*TDF*) could repair the extracellular damage induced by *SIV*. Here, we specifically investigate the timeframe that may be perfect for treatment initiation to achieve better outcomes and— how long this regimen could take to achieve full recovery. We also investigate whether the regimen exacerbates the detrimental effects of *SIV* on the brain to a certain extent. Lastly, we determine whether the improvement observed in the immune system, if it exists, is in parallel to the improvement in the intra-and-extracellular milieu of the brain.

Evaluation of extracellular free water content is one of the approaches to studying alterations in the extracellular environment (5–8). Here we use a multi-shell two-compartment model (9, 10), also known as a bi-tensor model (11–13), to obtain information about a fast-diffusing component, free water, in the extracellular space of rhesus monkeys using diffusion tensor images. At each voxel of the rhesus monkey brain, we separate components of extracellular space from those of intracellular space, consistent with previous studies (8, 14, 15), and compute the free water volume fraction (*FW-VF*) and the free water-eliminated diffusion properties of the tissues, such as the free water corrected fractional anisotropy (*FW*-*FA*), mean diffusivity (*FW*-*MD*), axial diffusivity (*FW*-AD), and radial diffusivity (*FW*-RD) (8). Greater free water volume has been identified in the early stages of neurological disorders such as schizophrenia, and researchers of extracellular water imaging have associated such increasing extracellular free water with the acute neuroinflammatory process (6, 16). Given that HIV is an inflammatory disease, the data from this study of extracellular water content are likely to answer the question in further details. We validate whether inflammation processes are likely at the core of free water volume changes by examining the fluctuations in free water volume during the initial phases of inflammatory SIVmac239 infection.

## 2 Materials and methods

### 2.1 Animal experimental setups and preparations

The experiments of this study were conducted at Beijing You An Hospital, the Capital Medical Hospital. The study received approval from the ethical committee of the Beijing Municipal Science and Technology Commission, the University of Science and Technology of China, and Wenzhou Medical University. The study complied with the code of ethics of the World Medical Association for animal experiments. The study involved ten male rhesus Monkeys of Chinese origin. We initially evaluated our animals to determine their health status. We conducted an indirect immunoassay (IFA) to identify and exclude the rhesus monkeys with simian immunodeficiency virus (*SIV*), Simian type D retrovirus (SRV), herpes B virus, or Simian T cell lymphotropic virus (STLV-1). All rhesus monkeys were in good health at the time of recruitment. We housed our rhesus Monkeys at the Institute of Laboratory Animal Sciences, Peking Union Medical College, while maintaining the optimal conditions—a temperature range of 16 to 26 degrees Celsius (°C), a humidity level between 40% and 70%, and a light/dark cycle of 12 hours each (light/dark =12hr/12hr). We gave the rhesus monkeys food twice a day without any restrictions on their diet, and we exposed them to unrestricted water access (water ad libitum).

### 2.2 Animal longitudinal experiments and designs

**Figure 1:**
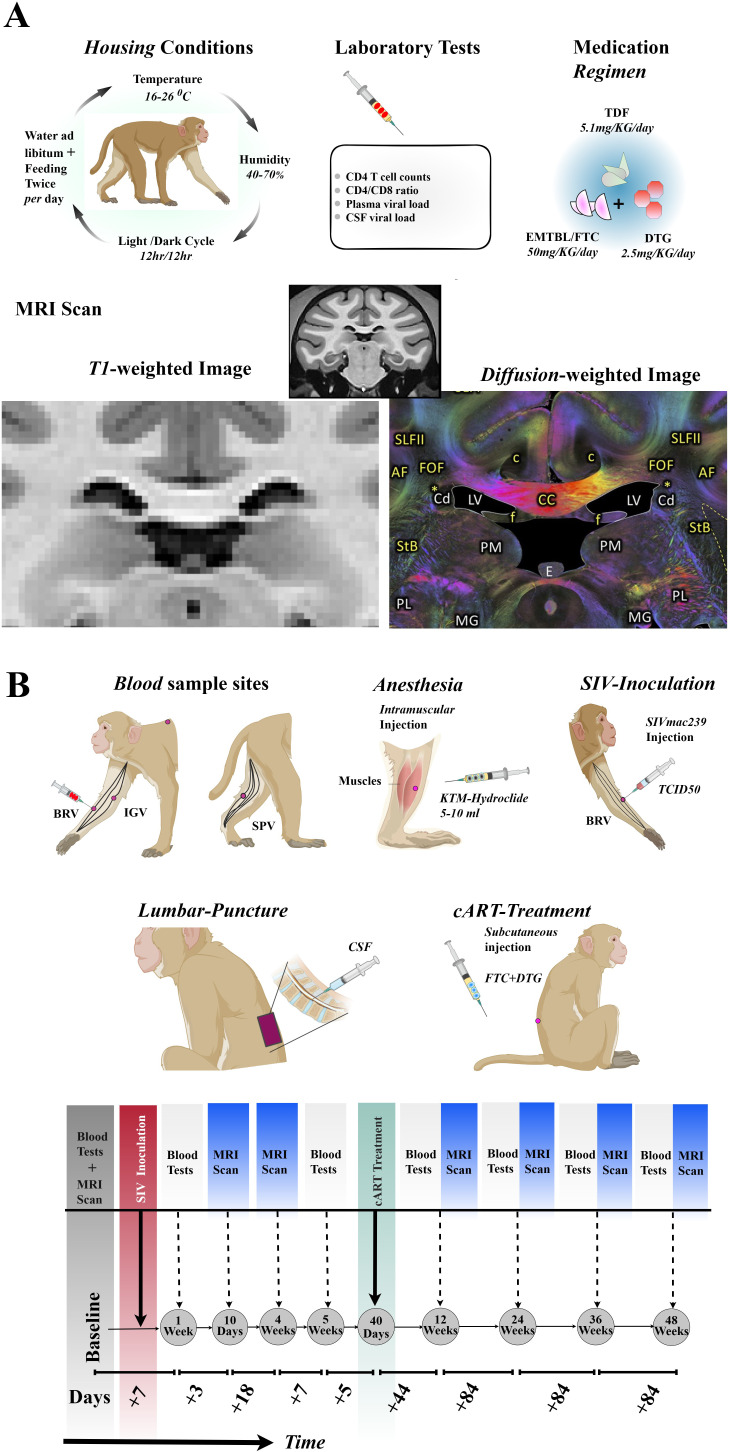
Experimental setups and designs. **(A)** Rhesus monkeys were housed in *16-26°C, 40-70 %* humidity, 12hr/12hr light/dark cycle, and fed twice a day with libitum. Laboratory tests including *CD4* T-cell count, *CD4/CD8* ratio, plasma and *CSF* viral load were examined before and after SIVmac239 infection. *5* out of *10* Chinese-origin rhesus monkeys were administered with *5.1 mg* of *TDF*, *50 mg* of *EMTBL/FTC*, and *2.5 mg* of *DTG*. Follow up examinations were conducted at 7 stages, with *MRI* Scanning involving T1-weighted images and diffusion-weighted images. **(B)** The SIVmac239 injection was administered through the brachial veins of the forelimb. The rhesus monkeys were anesthetized with an intramuscular injection of *5-10* ml of *KTM*-hydrochloride before the collection of *CSF* samples from lumbar puncture for *CSF*-*SIV* copy assays and before *MRI* scanning. Blood samples for immune level and disease progression assays were collected from the brachial (*BRV*) and inguinal (*IGV*) veins of the forelimbs, and the saphenous vein (*SPV*) of the hind limbs. Part of the *cART* (*FTC*+*DTG*) was administered via subcutaneous injection, while other doses were consumed through diet. The seven follow-up stages included baseline through day 336. TDF, tenofovir disoproxil fumarate; DTG, dotutegravir; EMTBL/FTC, the combination of emtricitabine; SIV, simian immunodeficiency virus; CSF, cerebrospinal fluid; KTM, Ketamine; MRI, magnetic resonance imaging.

**Figure 1** shows a full experimental setup and design, including regimen combinations, laboratory tests involved, housing conditions, and procedures during blood/CSF/MRI data collections. The details of the specific days or weeks on which each examination was performed are summarized in **Table 1**. Briefly, to accomplish our objective, which involves the identification of early inflammatory-related changes in the extracellular environment following SIVmac239 infection and the potential restorative effects of the *TDF*+EMTBL+*DTG* regimen, we designed and conducted a series of follow-up experiments for 48 weeks (336 days). We acquired MRI images at 7 stages, i.e., at the baseline, and on day 10, day 28, day 84, day 168, day 252, and day 336 following SIVmac239 infection (**Figure 1B**). MRI imaging closely followed the experiments for blood sample collection and assays, which were conducted at 7 stages as well, i.e., at the baseline, and on day 7, day 35, day 84, day 168, day 252, and day 336 after infection. Step by step description of the experiments is as follows. Briefly, at the baseline, i.e., before injecting rhesus monkeys with SIVmac239 dose, we set protocols for data collection. We collected blood samples from the forelimbs (specifically, from the brachial and inguinal veins) and the hindlimbs (specifically, from the saphenous vein). From the blood samples, we quantified the primary biomarkers of immunity levels. We first quantified the number of CD4 T-cells present in 1 μl of the blood collected from the same site for each monkey. We next quantified the number of CD8 T-cells present in 1 μl of the blood. We later calculated the ratio of the number of CD4 T-cells to the number of *CD8* T-cells in 1 μl of the blood— this ratio reflects the stability of the immune system and is commonly referred to as the *CD4/CD8* ratio. We then gave all monkeys intramuscular injections of anesthetic drugs, *Ketamine hydrochloride (5* -10 *ml)* —and scanned their brains with T1-and diffusion-weighted protocols.

**Table 1:** Schedule for experimental examinations.

Week	CD4 counts	CD8 counts	CD4/CD8 ratio	Weight	MRI	Plasma VL	CSF VL
W0	day 0	day 0	day 0	T1(W0)	T1 (day 0)	T1 (day 0)	T1 (day 2)
W1	day 7	day 7	day 7	–	T2 (day 10)	T2 (day 7)	–
W2	day 14	day 14	day 14	T2(W2)	–	–	T2 (day 14)
W5	day 34	day 34	day 34	T3(W4)	T3 (day 28)	T3 (day 35)	T3 (day 34)
W8	day 56	day 56	day 56	–	–	–	–
W12	day 90	day 90	day 90	T4(W12)	T4 (day 84)	T4 (day 90)	T4 (day 90)
W24	day 173	day 173	day 173	T5(W24)	T5 (day 168)	T5 (day 173)	T5 (day 173)
W36	day 256	day 256	day 256	T6(W36)	T6 (day 252)	T6 (day 256)	T6 (day 256)
W48	day 340	day 340	day 340	–	T7 (day 336)	T7 (day 340)	T7 (day 340)

#### 2.2.1 SIV239 infection and follow-up

We next gave the rhesus monkeys injections of SIVmac239 (i.e., a 50 fold half-tissue culture infective dose) through brachial veins. The TCID50 of SIVmac239 was later transformed to *SIV*. Seven (7) days later, we took blood samples from the monkeys. We also obtained CSF samples of the rhesus monkeys from the lumbar spineous space. We next quantified the number of copies of *SIV* virus in 1 ml of blood samples and CSF samples from macaques, representing the viral load in plasma and CSF, respectively. We used quantitative PCR (qPCR) for this test. The number of copies of the *SIV* provides information on disease progression. We then counted the number of CD4 T-cells and CD8 T-cells in 1 μl of blood samples to track the level of the immune system and response. Three (3) days later, we gave all monkeys intramuscular injections of anesthetic drugs, *Ketamine hydrochloride (5* -10 *ml)* —and scanned their brains with T1-and diffusion-weighted protocols. Follow-up MRI and blood experiments were carried out on day 28 (MRI only) and day 35 (blood and CSF only) before the cART initiation.

#### 2.2.2 cART intervention and follow-up

After forty (40) days of SIVmac239 infection (i.e., 4 weeks+5 days), we gave 5 of 10 rhesus monkeys suppressive cART regimen. We subcutaneously injected the rhesus monkeys with emtricitabine (FTC, 50 ml per day) and tenofovir disoproxil fumarate (*TDF*, 5.1 ml per day). We also mixed dotutegravir (*DTG*, 2.5 mg/kg daily) into their diet. After forty-four (44) days of suppressive cART (i.e., on the 84th day of SIVmac239 infection), we collected blood, CSF, and image samples from these animals and assessed the body’s response to the FTC+TDF+DTG regimen. We further investigated the impact of this regimen on intracellular and extracellular damage repair. Additional follow-up experiments were conducted on days168, 252, and 336 (post inoculation).

### 2.3 Macaque neuroimaging protocols

Neuroimaging of the brains of rhesus monkeys was performed on a Siemens 3T MRI Scanner (Siemens, Tim TRIO, and Germany Erlangen, Germany) at Beijing You An Hospital, the capital Medical hospital. The number of MRI scanning experiments conformed to the details shown in **Figure 1B**. We set protocols for the two imaging sequences—3D-T1-weighted anatomical and diffusion-weighted images. We used the following settings for 3D-T1-weighted imaging: TR/TE = 1,800 ms/4.12 ms, field of view (*FOV*) = 18.9 mm × 18.9 mm x 7.2 mm, Data matrix (matrix size) = 192 × 192 x 72, flip Angle = 90, slice thickness = 1 mm, and voxel size =0.984 mm × 0.984 mm × 1 mm; whereas we set the following parameters for diffusion-weighted imaging: Echo planar, 120 diffusion-encoded (60, b = 1,000 s/mm2; 60, b = 2,000 s/mm2), 2 references (b = 0 s/mm2), TR/TE = 5200 ms/100 ms, field of view (*FOV*) = 15.2 mm × 15.2 mm x 8.0 mm, Data matrix (matrix size) = 76 x 76 x 40, flip Angle = 900, slice thickness =2 mm, and voxel size =2 mm × 2 mm × 2 mm; time points = 122 volumes; oblique = 15.8720.

### 2.4 Processing of Macaque neuroimaging data

Macaque MRI data processing was in accordance with earlier studies (6, 17). We first preprocessed the data in the procedures that involve five steps. We began by extracting the monkey brains from the raw T1-WI and DWI data using three different approaches: (1) ‘3dSkullStrip’ tool with ‘monkey’ flag implemented in AFNI (AFNI, v23.1.08, https://afni.nimh.nih.gov/) (18); (2) ‘bet4animal’ tool implemented in FMRIB Software Library (Oxford, UK, FSL, v6.0.6.6, http://www.fmrib.ox.ac.uk/fsl/); and (3) ‘U-net model for brain extraction’ tool implemented in DeepBet-U-Net (DeepBetv1.0, https://github.com/HumanBrainED/NHP-BrainExtraction) (19). DeepBet extracts the brains of non-human primates using deep learning models. These models previously received initial training on human brains, and they were later updated with the brains of non-human primates by Wang et al. We further cross-trained one of the models with the brains of our rhesus monkeys. Here we used the ‘Site-All-T-epoch-36-update-with-Site-6-plus-7-epoch-09’ model, which was initially updated by Wang et al. with brains of 19 macaques from 13 sites specified by PRIDE-DE. Our two experts, Dr. BA. N and Dr. Y.L, conducted a thorough visual inspection for quality assessment of brain extraction performed by each of the three approaches. They then voted for the approach with the best performance on our data. Experts found DeepBet’s results sufficient for subsequent processing steps due to better smoothing and fine-area delineation (See **Supplementary Figure S1A** for the results of AFNI-3dSkullStrip and FSL-bet4animal, and **Supplementary Figure S1B** for the results of DeepBet-U-Net). We next corrected for eddy-current induced distortion and head-motion artifact using FSL’s ‘eddy-current’ tool (20). Before computing for water molecules (*FW*) diffusing freely in the extracellular space, we first fitted the DWI data using the standard FLS’s ‘DTI-FIT’ to obtain DTIFIT-derived raw *FA* maps. We then non-linearly registered the DTIFIT-derived raw *FA* map to a standard space defined by UWDTIRhesusWM template, using FSL’s registration tools (FLIRT/FNIRT). Then, the multi shell DWIs data encoded in 60 directions for b = 1000 s/mm2 and 60 directions for b = 2000 s/mm2 were again put into Python code to calculate *FW*, and *FA* and *MD* corrected for *FW* (*FW*-*FA*, *FW*-*MD*). Briefly, our code uses a bi-tensor model to split a single voxel information into extracellular *FW* and tissue compartments. We particularly used an optimized version of the model proposed by Hoy et al. (11), originally described by Pasternak et al. (21) in its expanded form: $S\left(g,b\right)=S_{0}\left(1-f\right)e^{-bg^{T}Dg}+S_{0}fe^{-bD_{iso}}$ Where g and b are diffusion gradient direction and weighted, S(g,b) is the diffusion-weighted signal measured, $S_{0}$ is the signal in a measurement with no diffusion weighting, D is the diffusion tensor, f is the volume fraction of the free water component, and $D_{iso}$ is the isotropic value of the free water diffusion(normally set to $3.0\;times\;10^{-3}mm^{2}s^{-1}$ The generated new metrics, *FW-VF*, *FA-FW*, and *MD-FW*, were further transformed into standard space using the transformation matrix obtained during the transformation of the DTI-derived raw *FA* map to a standard space defined by UWDTIRhesusWM template. Our two experienced neurologists (X.W. and B.N., with 10 and 6 years of experience, respectively) visually inspected our data for registration quality validation. It is important to note that the UWDTIRhesusWM template used here is rhesus macaque white matter based, developed using high-quality brain scans of 271 rhesus monkeys (https://www.nitrc.org/projects/rmdtitemplate/) (22). The template accounts for signal and spatial variability across species. It also addresses the previously identified challenges of low signal contrast, low signal-to-noise ratio, low sharpness, and low visibility of fine white matter structures and spatial features of other templates (22).

## 3 Statistical analysis

We performed statistical analyses for metrics from the MRI images and measures from blood and CSF samples on R v4.2.0 (23). Briefly, we applied mixed-effect models (ANOVA models) to determine the effects of *SIV* and time, and the impact of FTC/EMTBL+*TDF*+ *DTG* regimen on the brain, particularly on the extracellular environment as quantified by *FW*, and on the WM integrity as quantified by *FW*-*FA* and *FW*-*MD* metrics. Prior to these analyses of effects, we checked if our data met the assumption of normality using Shapiro Wilk Test and the assumption of homogeneity of variance using Levene’s Test. The data that met these assumptions received the ANOVA tests, followed by independent t-test (student t-test) or paired sample t-test in the *post-hoc* analyses. Otherwise, the data violated these assumptions underwent the Welch’s test and Wilcoxon Rank sum Test (Mann Whitney U Tests). Of note is that, for analyses of effects (ANOVA), the Between-Subject factor had two levels, *SIV*+cART- and *SIV*+cART+. The *SIV*+cART- denotes rhesus *SIV*-infected macaques without suppressive cART, whereas the *SIV*+cART+ denotes rhesus *SIV*-infected macaques with initiated suppressive cART. Within-subject factor for our analyses of effects (ANOVA) was time with 7 levels (7 time-points). The seven levels were the baseline, day 10, day 28, day 84, day 168, day 252, and day 336 following the SIVmac239 infection. We performed further statistical tests for identification of potential relationship between clinical measures (CD4 T-cells, *CD4/CD8* ratio, viral load) and MRI metrics of extracellular disruption and microstructural tissue damage. To this end, we used Pearson’s correlation method and Spearman’s correlation method, depending on whether the data met the assumptions of normality, linearity, and homoscedasticity. Statistical errors that arose due to multiple comparisons were corrected using false discovery rate (FDR) technique. Statistical measures for the analyses of effects and correlations involved β coefficients, F, r, and P values—with cut-off set at p <.05 for significance.

## 4 Results

### 4.1 Macaque’s demographics

Briefly, all 10 rhesus monkeys were male, of Chinese origin, and aged 3.8 ± 0.3 years. At the time of recruitment, the rhesus monkeys were all healthy, with an average weight of 4.72 ± 0.58 kg. The ranges of their CD4 T-cell count, CD8 T-cell count, and *CD4/CD8* ratio were 566—2342, 235—881, 0.97—3.0, respectively.

### 4.2 CSF and blood assays after SIV239 injection

Before injections of TCID5O for SIVmac239 infection, the average scores of CD4 T-cell count, CD8 T-cell count, and *CD4/CD8* ratio were 1120.79 ± 514.25, 605.81 ± 201.47, and 1.96 ± 0.78, respectively. After 7 days of TCID5O injection, the SIVmac239 transformed into *SIV* infection. The number of SIV copies per ml increased from 0 to 6.89 ± 0.44 on day 7 (P < 0.0001, in plasma, **Table 2**) and continued to rise to 6.92 ± 0.40 on day 14 (P < 0.0001, in CSF, **Table 2**) and to 7.17 ± 0.51 on day 34 (P < 0.0001, in plasma). For CD4 T-cells (**Table 3**), there was no significant change in their number by day 7 (1187.68 ± 468.95 cells/µL, paired t-test, *P* = 0.6000). A statistically significant change in CD4 T-cells was first detected on day 14; the CD4 T-cells dropped to 756.29 ± 404.44 cells/µL (*P* = 0.0022). As the disease progressed to day 34, the CD4 T-cells continued to remain below < 1000 (837.66 ± 351.59 cells/µL, day 34, *P* = 0.0495). The CD8 T-cells, on the other hand, began to manifest change by day 7; they increased from 605.81 ± 201.47 cells/µL at baseline to 871.01 ± 467.53 cells/µL, although this increase was not statistically significant (*P* = 0.0907). By day 14, the change in CD8 T-cells was more evident, increasing to 1012.38 ± 237.49 cells/µL, *P* = 0.0010). This increase in CD8 T-cells persisted through day 34 (1114.42 ± 234.84 cells/µL, *P* = 0.0009) as the viral infection continued. For the CD4/CD8 ratio, we started to observe a trend of decline by day 7; the ratio decreased to 1.65 ± 0.93 from 1.96 ± 0.78 at baseline. However this change was not statistically significant (*P* = 0.1782). An intensified change in the CD4/CD8 ratio for our rhesus monkeys manifested on day 14 (dropped to 0.78 ± 0.40, *P* < 0.0001)—and this reduction in CD4/CD8 ratio progressed as the disease advanced (0.77 ± 0.36, P < 0.0001, day 34). These results suggest that a significant reduction in immunity levels—CD4 T-cells and CD4/CD8 ratio—begins as early as the first 14 days (two weeks) of infection and persists as the disease advances. The reduction in immunity levels and the increase in CD8 T-cell proliferation and differentiation coincided with a rise in the number of SIV copies.

**Table 2:** SIV viral record in rhesus monkeys after SIV239 infection and suppressive cART drugs.

Comparison of SIV copies before and After SIV239 Infection (Paired T-Test, the comparison against T1 (Baseline))																
			T1	T2						T3						
			Mean±SD	Mean±SD		*P*		t		Mean±SD			*P*		t	
Plasma VL			2±0	6.89±0.4407		6.12422E-11		-35.0921		7.17±0.5136			1.46E-10		-31.8368	
CSF VL			2±0	6.92±0.3964		2.23852E-11		-39.2702		5.71±0.4554			9.65E-10		-25.7600	

Comparison of SIV copies before and After cART (Paired T-Test, the comparison against T1 (Baseline))																
		T3	T4			T5				T6			T7			
		Mean±SD	Mean±SD	*P*	t	Mean±SD	*P*		t	Mean±SD	*P*	t	Mean±SD	*P*	t	
Plasma VL	cART-	2±0	6.72±0.6519	0.0001	-16.1841	6.99±0.7633	0.0001	-14.6247		6.96±0.9535	0.0003	-11.6407	7.14±0.6073	0.0001		-15.0717
	cART+	2±0	3.34±1.3275	0.0873	-2.2538	2.58±0.7997	0.1778	-1.6329		2.17±0.3891	0.3739	-1.0000	2.70±1.0228	0.2017		-1.5260
CSF VL	cART-	2±0	5.06±0.6062	0.0003	-11.3020	3.73±1.6090	0.0740	-2.4042		4.56±0.7481	0.0016	-7.6400	5.00±0.5106	0.0013		-11.7519
	cART+	2±0	2.95±1.3856	0.2000	-1.5331	2.28±0.6172	0.3739	-1.0000		2±0	-	-	2±0	-		-

Comparison of SIV copies before and After cART (Paired T-Test, comparison against T3 (5 days before cART initiation))																
		T3	T4			T5				T6			T7			
		Mean±SD	Mean±SD	*P*	t	Mean±SD	*P*		t	Mean±SD	*P*	t	Mean±SD	*P*	t	
Plasma VL	cART-	6.97±0.5311	6.72±0.6519	0.4401	0.8564	6.99±0.7633	0.9282		-0.0960	6.96±0.9535	0.9957	0.0057	7.14±0.6073	0.5349	-0.6990	
	cART+	7.38±0.4543	3.34±1.3275	0.0016	7.6217	2.58±0.7997	0.0008		9.0341	2.17±0.3891	0.0001	18.2046	2.70±1.0228	0.0005	10.1830	
CSF VL	cART-	5.57±0.4684	5.06±0.6062	0.1959	1.5507	3.73±1.6090	0.0461		2.8555	4.56±0.7481	0.0162	3.9936	5.00±0.5106	0.0249	4.1848	
	cART+	5.85±0.4437	2.95±1.3856	0.0043	5.8330	2.28±0.6172	0.0010		8.6821	2.00±0.0000	4.15E-05	19.4125	2.00±0.0000	4.15E-05	19.4125	

**Table 3:** Immunity Levels Before and After SIV239 Infection and Suppressive cART Drugs.

Immunity Levels Before and After SIV239 Infection (Paired T-Test, the comparison against Baseline)										
	Baseline	Day7			Day14			Day34		
	Mean±SD	Mean±SD	*P*	t	Mean±SD	*P*	t	Mean±SD	*P*	t
CD4	1120.79±514.25	1187.68±468.95	0.6000	-0.54	756.29±404.44	0.0022	4.23	837.66±351.59	0.0495	2.27
CD8	605.81±201.47	871.01±467.53	0.0907	-1.89	1012.38±237.49	0.0010	-4.78	1114.42±234.83	0.0009	-4.86
CD4/CD8 ratio	1.96±0.78	1.65±0.93	0.1782	1.46	0.78±0.40	0.0001	6.66	0.77±0.36	0.0001	6.25

Immunity Levels Before and After Suppressive cART Drugs (Paired T-Test, comparison against baseline)																	
		Baseline	Day56			Day90			Day173			Day256			Day340		
		Mean±SD	Mean±SD	*P*	t	Mean±SD	*P*	t	Mean±SD	*P*	t	Mean±SD	*P*	t	Mean±SD	*P*	t
CD4	cART-	1249.01±687.63	753.65±278.05	0.0688	2.47	626.71±273.24	0.0365	3.09	745.97±283.02	0.0943	2.18	393.45±202.53	0.0451	2.88	489±384.1	0.0147	4.11
	cART+	992.57±284.77	722.1±242.61	0.1402	1.84	721.05±261.36	0.1459	1.80	760.53±258.71	0.0868	2.26	770.02±237.15	0.1322	1.89	936.6±488	0.6847	0.44
CD8	cART-	709.12±155.24	774.36±222.61	0.6479	-0.49	756.26±265.10	0.7584	-0.33	1002.59±256.83	0.0894	-2.23	580±252.84	0.4316	0.87	487.2±154.85	0.0267	3.42
	cART+	502.5±201.35	527.24±125.96	0.8133	-0.25	622±268.97	0.4995	-0.74	556.13±181.66	0.6433	-0.50	539.34±141.46	0.7662	-0.32	486.6±149.49	0.8759	0.17
CD4/CD8 ratio	cART-	1.73±0.74	1.07±0.61	0.0038	6.03	0.91±0.47	0.0039	5.99	0.83±0.50	0.0061	5.30	0.97±0.89	0.0177	3.89	1.1±0.97	0.0819	2.31
	cART+	2.2±0.81	1.4±0.48	0.0123	4.34	1.19±0.37	0.0095	4.67	1.46±0.69	0.0035	6.15	1.45±0.47	0.0153	4.07	1.9±0.56	0.0708	2.45

Immunity Levels Before and After Suppressive cART Drugs (Paired T-Test, comparison against Day34)																	
		Day34	Day56			Day90			Day173			Day256			Day340		
		Mean±SD	Mean±SD	*P*	t	Mean±SD	*P*	t	Mean±SD	*P*	t	Mean±SD	*P*	t	Mean±SD	*P*	t
CD4	cART-	932.78±427.06	753.65±278.05	0.2454	1.36	626.71±273.24	0.0833	2.30	745.97±283.02	0.3200	1.13	393.45±202.53	0.0568	2.65	489±384.1	0.0343	3.16
	cART+	742.53±270.44	722.1±242.61	0.8937	0.14	721.05±261.36	0.8726	0.17	760.53±258.71	0.9120	-0.12	770.02±237.15	0.8539	-0.20	936.6±488	0.5016	-0.74
CD8	cART-	1166.18±270.76	774.36±222.61	0.0943	2.18	756.26±265.10	0.1303	1.90	1002.59±256.83	0.2698	1.28	580±252.84	0.0035	6.18	487.2±154.85	0.0039	5.99
	cART+	1062.66±209.93	527.24±125.96	0.0088	4.78	622±268.97	0.0473	2.83	556.13±181.66	0.0190	3.80	539.34±141.46	0.0088	4.78	486.6±149.49	0.0208	3.70
CD4/CD8 ratio	cART-	0.84±0.51	1.07±0.61	0.0170	-3.94	0.91±0.47	0.3845	-0.98	0.83±0.50	0.9454	0.07	0.97±0.89	0.5406	-0.67	1.1±0.97	0.3318	-1.10
	cART+	0.69±0.15	1.4±0.48	0.0100	-4.61	1.19±0.37	0.0080	-4.91	1.46±0.69	0.0358	-3.11	1.45±0.47	0.0089	-4.76	1.9±0.56	0.0034	-6.23

### 4.3 CSF and blood assays after suppressive cART drugs

**Figure 2:**
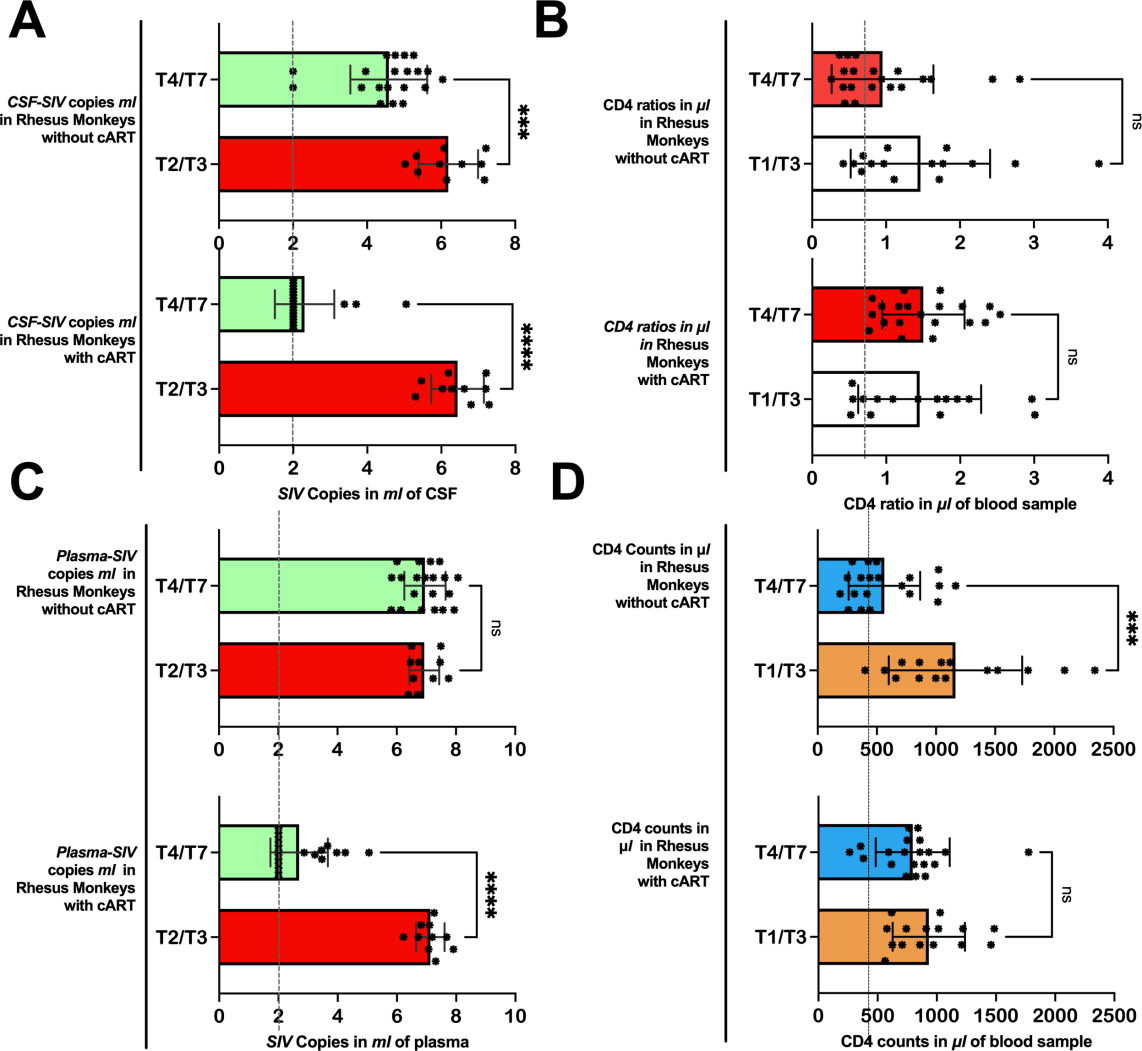
Immunity levels and *SIV* viral copies as the disease progresses in Chinese-origin rhesus monkeys with and without treatment. In 5 out of 10 rhesus monkeys, the *cART* treatment was introduced before T4 (at day 40). In rhesus monkeys without treatment, the number of *SIV* copies in ml of *CSF* **(A)** and plasma **(C)** remained relatively higher from T4 through T7, while those with treatment achieved viral suppression. Similarly, those with treatment succeeded in improving or maintaining their *CD4/CD8* ratio **(B)** and *CD4* T-cell counts **(D)** after treatment, but those without treatment continued to have relatively low *CD4* ratios and *CD4* T-cell counts. Also see **Supplementary Table S1** for further statistics. CSF-SIV, cerebrospinal fluid- simian immunodeficiency virus; T2/T3, time point including T2 and T3; T4/T7, time points including T4, T5, T6, and T7. ***P < 0.001, ****P < 0.0001. The expanded form of “ns” is a non-significant value at a threshold of 0.05, in other words, P > 0.05.

On day 40 of SIVmac239 infection, 5 of 10 rhesus monkeys were initiated by injection and through diet with suppressive cART drugs [FTC/EMTBL+*TDF*+ *DTG*]. Five days before cART initiation (day 40 of SIV infection), these specific five rhesus monkeys had an average CD4 T-cell count of 742.53 ± 270.44, a CD8 T-cell count of 1062.66 ± 209.93, and a CD4/CD8 ratio of 0.69 ± 0.15. Their average number of *SIV* copies per *ml* was 7.38 ± 0.45 in plasma and 5.85 ± 0.44 in CSF. After treatment, the viral suppressive effects of our regimens began to manifest within the first 44 days of progressive cART (see **Table 2**, bottom panel). By day 44(50) of the suppressive drug treatment, the number of SIV copies significantly declined; the number of plasma *SIV* copies reduced by 75.09%, dropping from 7.38 ± 0.45 to 3.34 ± 1.33, *P* = 0.0016). The SIV copies in CSF dropped as well from 5.85 ± 0.44 to 2.95 ± 1.39 (*P* = 0.0043). Our drug suppressive effects continued progressively over time. In short, 60% (3 of 5), 80% (4 of 5), and 100% (5 of 5) of rhesus macaques achieved a total virosuppression by day 44, day 128, and day 212, respectively. For immunity levels, we detected the immune-boosting effects of our drugs by day 16 of suppressive drug treatment (see **Table 3**). The CD4/CD8 ratio of the treated rhesus monkeys increased from 0.69 ± 0.15 to 1.4 ± 0.48 (*P* = 0.0100) by day 16 of suppressive drug treatment and continued to improve, reaching 1.9 ± 0.56 by day 212 (*P* = 0.0034). Meanwhile, the number of CD8 T cells was significantly reduced to 527.24 ± 125.96 (P = 0.0088) by day 16 and to 486.6 ± 149.49 (*P* = 0.0208) by day 212, from 1062.66 ± 209.93 before cART initiation. (See **Tables 2**, **3** for statistics on the progression of immunity levels and SIV viral copies as the disease progresses, as well as with cART intervention; also see **Figure 2**; **Supplementary Table S1** for overall comparisons before and after treatment).

On the other hand, the rhesus monkeys (5 out of 10) that did not receive cART intervention on day 40 continued to have elevated SIV copies on later days, similar to the levels seen on day 35 or earlier. On day 35, the plasma levels of SIV copies were 6.97 ± 0.53 cells/µL (*P* = 0.4401 vs. Baseline) and persisted on the follow-up days: day 90 (6.72 ± 0.65 cells/µL, *P* = 0.4401 vs. day 34), day 173 (6.99 ± 0.76 cells/µL, *P* = 0.9282 vs. day 34), day 256 (6.96 ± 0.95 cells/µL, *P* = 0.9957 vs. day 35), and day 340 (7.14 ± 0.61 cells/µL, *P* = 0.5349 vs. day 34). These untreated rhesus monkeys continued to experience severely worsened immunity levels as the disease advanced. Their CD4/CD8 ratio remained low(< 1) during the early days of infection, i.e., 0.76± 0.49 on day 14 (*P* = 0.0027 vs. baseline) and 0.84± 0.51 on day 34 (*P* = 0.0053 vs. baseline) —and continued to be low in later days: 1.07 ± 0.61 on day 56 (*P* = 0.0038 vs. baseline; *P* = 0.0170 vs. day 34), 0.91 ± 0.47 on day 90 (*P* = 0.0039 vs. baseline; P = 0.3845 vs. day 34), 0.83 ± 0.50 on day 173 (P = 0.0061 vs. baseline; *P* = 0.9454 vs. day 34), 0.97 ± 0.89 on day 256(*P* = 0.0177 vs. baseline; *P* = 0.5406 vs. day 34), and 1.1 ± 0.97 on day 340 (*P* = 0.0819 vs. baseline; *P* = 0.3318 vs. day 34). The number of CD4 T-cells declined progressively (below 1000) from the baseline (1120.01 ± 687.63) as the disease advanced. By day 34, the CD4 T-cells dropped to 932.78± 0.427(*P* = 0.0974 vs. baseline). After day 40 (when those in the other group were receiving cART), the CD4 T-cells of the untreated rhesus monkeys continued to decline to 753.65± 278.05 (*P* = 0.0688 vs. baseline; *P* = 0.2454 vs. day 34), 626.71 ± 273.24 (*P* = 0.0365 vs. baseline; *P* = 0.0833 vs. day 34), 745.97 ± 283.02 (*P* = 0.0943 vs. baseline; *P* = 0.3200 vs. day 34), 393.45 ± 202.53 (*P* = 0.0451 vs. baseline; *P* = 0.0568 vs. day 34), and 489 ± 384.1 cells/µL (*P* = 0.0147 vs. baseline; *P* = 0.0343 vs. day 34) by day 56, day 90, day 173, and day 340, respectively.

### 4.4 Animal weights before and after SIV239 injection or cART

Before the injection of TCID5O, the average weight rhesus monkeys was 4.72 ± 0.58 kg. After 14 days of infection, they lost 6% of their weight (from 4.72 ± 0.58 to 4.45 ± 0.55 kg, *P* = 0.0145, Paired t-test, see **Supplementary Table S2**). By day 28 of progressive diet, they regained the 6% of the lost weight (to 4.68 ± 0.58, *P* = 0.6882). Over time, the weight continued to increase (to 4.81 ± 0.781 by day 96). We did not find strong evidence of group effects on weights after the cART intervention (P > 0.5); both rhesus monkeys, with and without cART, gained weight over time. However, those who did not receive cART intervention demonstrated a more pronounced weight gain trend than those who received it, especially from days 14 to 28 (cART-, 4.64 ± 0.72 to 4.9 ± 0.75, *P* = 0.0004; cART+, 4.26 ± 0.26 to 4.46 ± 0.26, *P* = 0.0217) and days 84 to 168 (cART-, 4.92 ± 0.65 to 5.28 ± 0.75, *P* = 0.0288; cART+, 4.18 ± 0.29 to 4.33 ± 0.49, *P* = 0.2344).

### 4.5 Early brain alterations in the extracellular spaces and diffusion properties in 10 rhesus monkeys, within the first 28 days of SIV239 infection

As early as within the first 28 days of SIV239 infection, both the extracellular water volumes and the diffusion of fiber tissues of the rhesus monkeys demonstrated changes (see **Table 4**). Briefly, free water volumes increased significantly for extracellular spaces surrounding the fiber tissues of the internal capsule (anterior, posterior and retrolenticular limbs), corona radiata (right and left anterior), thalamic radiata (right posterior), sagittal striatum (right), external capsule (right and left), longitudinal fasciculus (right and left superior), uncinate fasciculus(right), prefrontal WM(right and left of both dorsal and ventral parts), frontal gyrus WM(left inferior), temporal gyrus WM(left superior), and cingulate WM(left anterior). This increase was more pronounced (survived FDR-corrections for multiple comparisons) in the internal capsule (left anterior limb), corona radiata (left anterior), thalamic radiation (right posterior), external capsule (left), longitudinal fasciculus (left superior), uncinate fasciculus (right), and prefrontal WM (left ventral). Exceptionally, for the cerebellar peduncles (right and left, inferior and superior) and the tapetum (right), the extracellular FW volume decreased. An intensified decrease (surviving FDR corrections for multiple comparisons) was observed in the right inferior cerebellar peduncle and the left superior cerebellar peduncle.

**Table 4:** Significant regions of FW-VF and FW-FA with time point and Group.

Time Range	Region Label	Region Name	p-value
*FW-VF*			
Before Treatment	10	Medial Lemniscus - Left	0.0166
	22	Retrolenticular Limb of the Internal Capsule - Left	0.0452
	59	Central Tegmental - Left	0.0087
After Treatment	32	Sagittal Striatum - Left	0.0284
	58	Central Tegmental - Right	0.0080
*FW-FA*			
Before Treatment	49	Anterior Commissure	0.0494
	52	Ventral Prefrontal WM - Right	0.0381
	70	Adjacent Thalamus WM - Left	0.0259
	76	Anterior Cingulum WM - Left	0.0064
After Treatment	9	Medial Lemniscus - Right	0.0262
	32	Sagittal Striatum - Left	0.0308
	40	Stria Terminalus - Left	0.0422
	47	Tapetum - Right	0.0191
	53	Ventral Prefrontal WM - Left	0.0310
	58	Central Tegmental - Right	0.0454

Meanwhile, early alterations to the diffusion properties, i.e., FA and MD corrected for FW, were also observed within this 28-day period of SIV239 infection. Briefly, the values of FW-FA decreased progressively in several fiber tissues, particularly in the corticospinal tract (left), corona radiata (right superior), cingulum (left superior), central tegmental (left), pyramidal tracts, and temporal gyrus WM (left superior). The decrease was more severe (survived FDR-corrections for multiple comparisons) in the left central tegmental. Meanwhile, two fibers, the cerebellar peduncle (middle) and uncinate fasciculus (right and left), displayed abnormal pattern of increased *FW*-*FA* values in the early days of *SIV* infection, with the uncinate fasciculus fiber tissues demonstrating an intensified increase (survived FDR-corrections for multiple comparisons). Most fibers of rhesus monkeys demonstrated changes in FW-MD values— at these early stages of SIV infection. Notably, there was an increase in FW-MD values in two major fibers— the cerebellar peduncle (right and left inferior, and left superior) and the internal capsule (retrolenticular limb), whereas decreased FW-MD values were detected in 15 other fiber tissues. Such fiber tissues with decreased FW-MD values include the corpus callosum (the genu, body and splenium), fornix, internal capsule (right and left anterior limb, and left posterior limb), corona radiata (right and left anterior), sagittal striatum (left), external capsule (right and left), Stria terminalus (right and left), uncinate fasciculus (right), commissure (anterior), prefrontal WM(both right and left, dorsal and ventral), pyramidal tracts frontal gyrus WM(right and left inferior), temporal gyrus WM (right middle), midbrain WM(right), and cingulum WM(right and left anterior). 70% of these WM fiber structures had an intensified FW-MD decrease (survived FDR correction for multiple comparisons) (See **Table 4**).

### 4.6 Extracellular content of rhesus monkeys following *cART* intervention (*TFG+TDF+DTG*)

**Figure 3:**
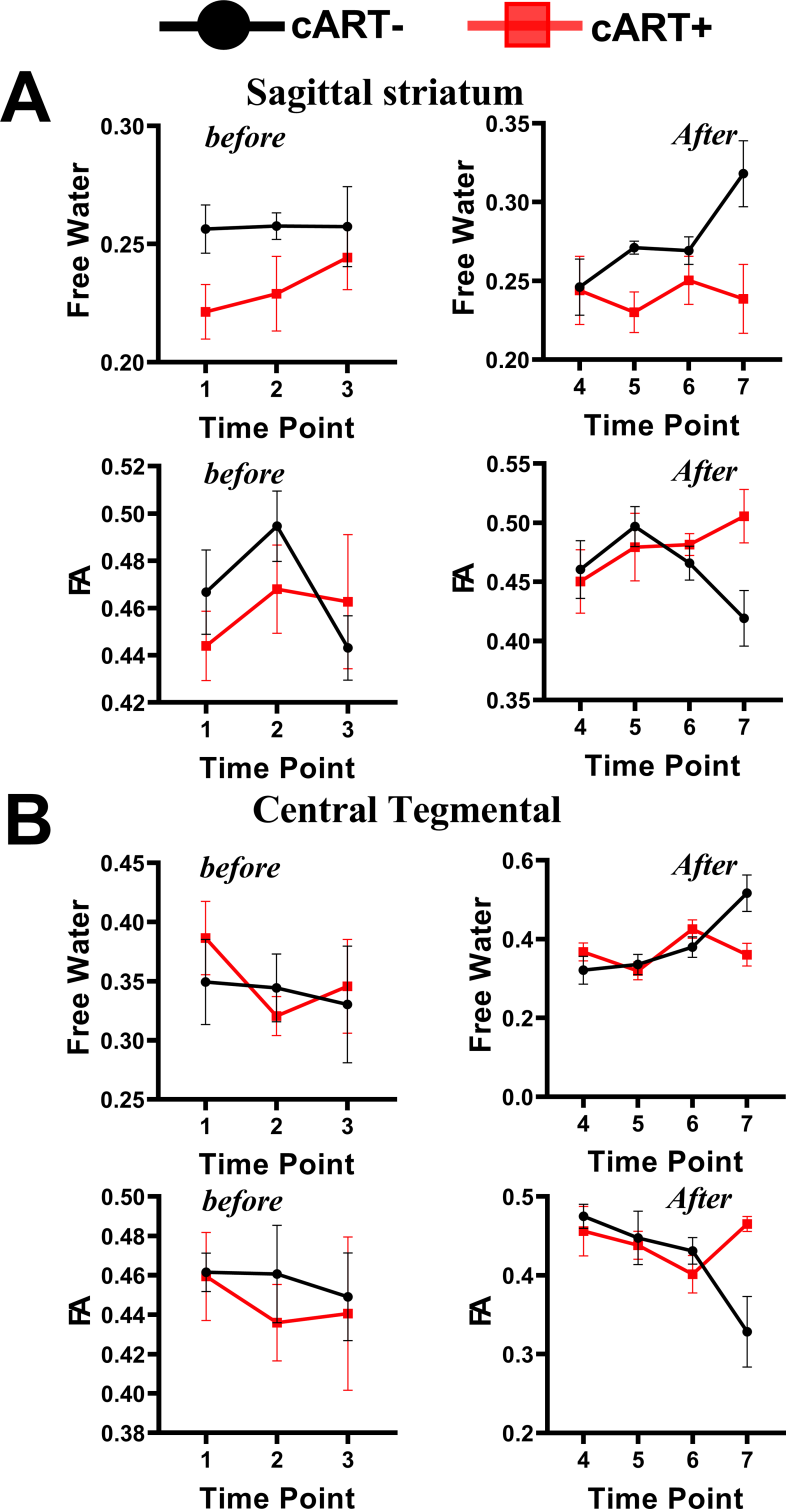
Interactive effects of *cART* regimen and time on extracellular *FW-VF* and *FW-FA*. The Chinese-origin rhesus monkeys exhibit interactive effects of *cART* over time, such that those without *cART* demonstrated a significant increase in *FW-VF* and a reduction in *FW-FA* over time across the sagittal striatum **(A)** and central tegmental area **(B)**, particularly at T7. *FW-FA*, free water corrected *FA* values; T7, time point 7; *cART*, combination of antiretroviral therapy.

**Figure 4:**
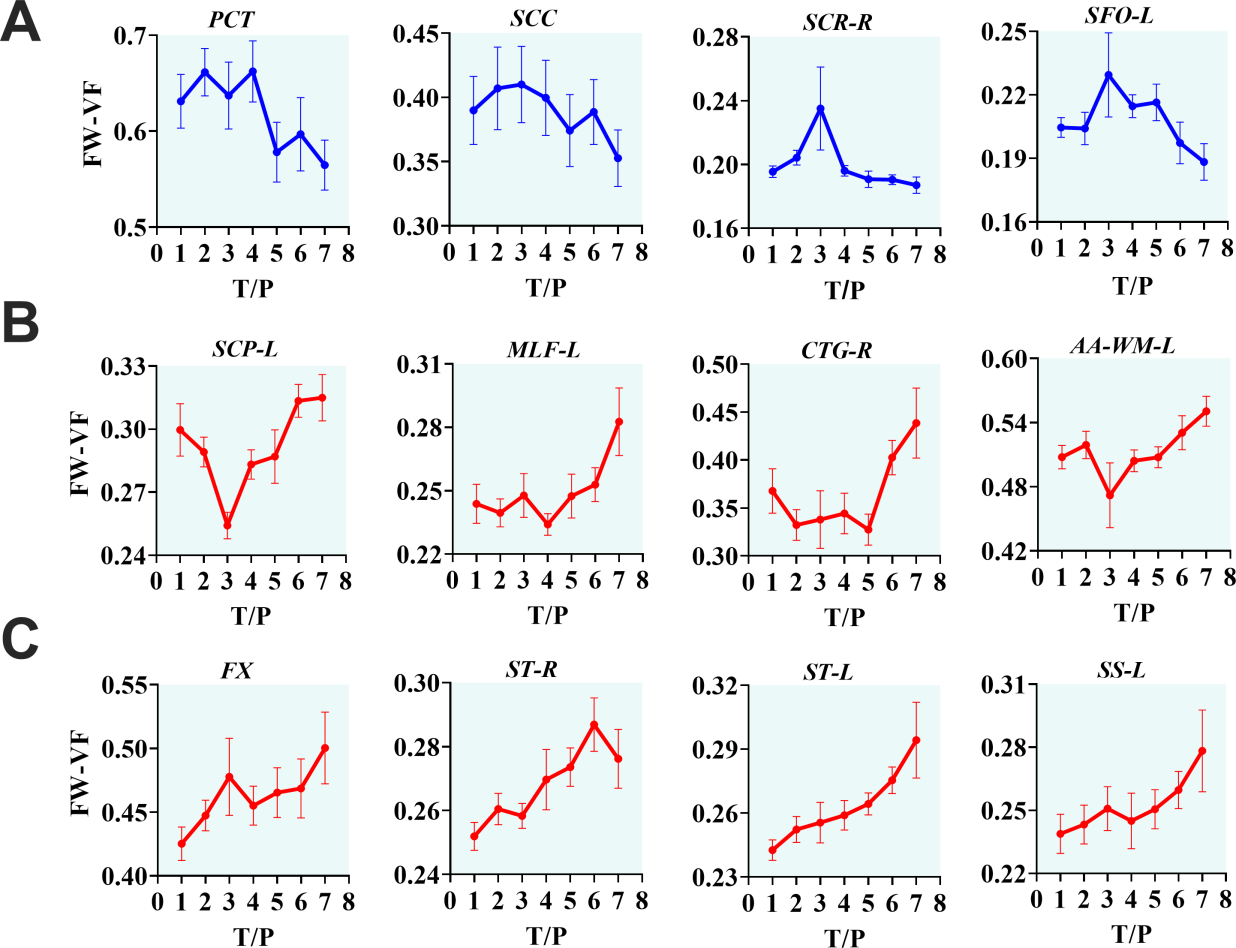
The progressive effects of SIVmac239 on extracellular environment over time, independent of *cART* status. The longitudinal effects of *SIV* infection on the extracellular environment appear in three forms. They may manifest as decreasing *FW-VF* in some fiber tissues, especially in *PCT*, *SCC*, *SCR-R*, and *SFO-L*, with intermediate fluctuations before descending to low levels of *FW-VF* **(A)**. However, in the majority of fiber tissues, changes in the extracellular space induced by inflammatory activities of *SIV* result in increasing *FW-VF*, with two noticeable trends **(B, C)**, but leading to the same endpoint. PCT, pontine crossing tract; SCC, splenium of corpus callosum; SCR-R, right superior corona radiata; SFO-L, left superior fronto-occipital fasciculus; SCP-L, left superior cerebellar peduncle; MLF-L, left medial longitudinal fasciculus; CTG-R, right central tegmental; AA-WM-L, left adjacent amygdala white matter; FX, fornix; ST-R, right stria terminalus; ST-L, left stria terminalus; SS-L, left sagittal striatum.

From the analysis of interactive effects, we found MRI evidence of changes in the extracellular environment in rhesus monkeys following SIVmac239 infection and cART intervention. Briefly, there were interactive effects of cART treatment and time on the FW-VF of the extracellular spaces crossed by two fiber tracts—the left Sagittal Striatum (SS-L) and the right Central Tegmental Fiber Tract (CTFT-R) (**Figure 3**, also see **Table 4**). The *FW-VF* of these fiber tracts increased significantly over time for the rhesus monkeys that received *cART* intervention. Such an increase was significantly slowed in those who received a *TFG+TDF+DTG* regimen on day 40. It is important to highlight that, prior to *cART* intervention on day 40, all rhesus monkeys (from all groups/sets) had a similar trend of increasing *FW-VF*, with minimal variations (nearly negligible). The differences in extracellular *FW-VF* between the two groups of rhesus monkeys began to unfold after day 40 of *cART* intervention. The greatest differences in *FW-VF* (*cART*- vs. *cART*+, see **Figure 3**; **Supplementary Table S8** for statistics) were identified on day 336 (time point 7) of *MRI* scanning (296 days after c*ART* intervention).The analyses of independent effects revealed independent effects of time (arguably reflecting the impact of *SIV239* infection) on the extracellular *FW-VF* in *SIV*-infected rhesus monkeys, independent of cART status (see **Figure 4**, also see **Supplementary Table S3**). Fourteen (14) fiber tracts were identified with independent effects of time. Two patterns of *FW-VF* attenuation were identified within these fibers. The first pattern of *FW-VF* disruption was characterized by a continuous and progressive increase in extracellular *FW-VF* over time. This pattern was observed in eight(8) fiber tissues, including the superior cerebellar peduncle, medial longitudinal fasciculus, central tegmental, adjacent amygdala WM, fornix, Strial terminalus right, Strial terminalus left, and sagittal striatum. The second pattern of *FW-VF* disruption unfolded as a progressive decline in *FW-VF* and was more evident in four fiber tracts, i.e., the pontine crossing tract, the splenium of the corpus callosum, the superior corona radiata, and the superior fronto-occipital fasciculus. While we generally observed these two patterns of changes over time, we also found time-specific, region-specific, sharp, and excessive changes in FW-VF in the early days of SIV239 infection. In particular, there was a sharp increase in FW-VF in the extracellular spaces surrounding the superior corona radiata, fornix, and superior fronto-occipital fasciculus on day 28 following the infection of SIVmac239. Conversely, a sharp decrease in FW-VF was observed in the extracellular spaces surrounding the superior cerebellar peduncle and adjacent amygdala WM.

### 4.7 FW-corrected diffusion properties of rhesus monkeys following cART intervention (TFG+TDF+DTG)

**Figure 5:**
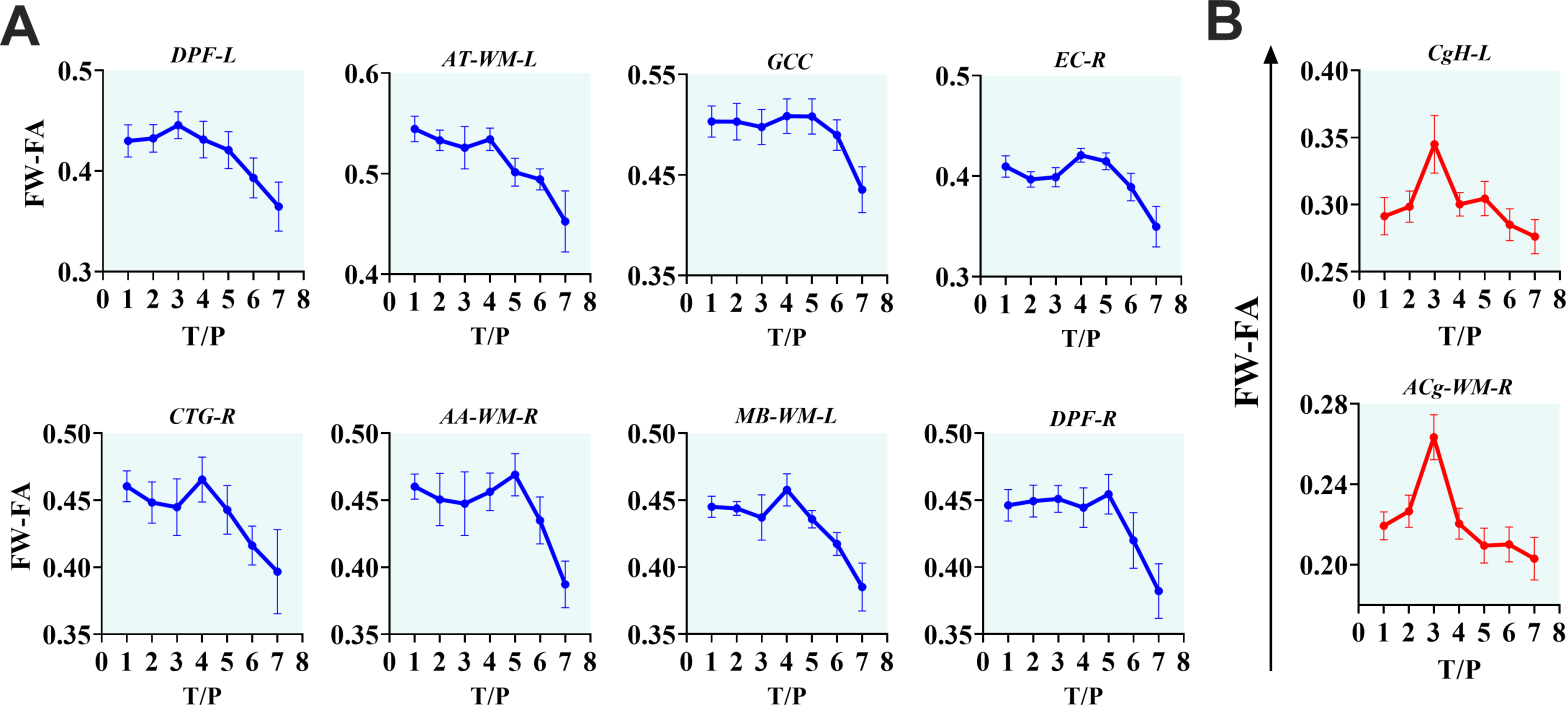
The progressive effects of SIVmac239 on *FW-FA* over time, independent of *cART* status. **(A, B)** The longitudinally independent effects of *SIV* infection on *FW-FA* are more apparent within fiber tissues of *DPF-L*, *AT-WM-L*, *GCC*, *EC-R*, *CTG-R*, *AA-WM-R*, *MB-WM-L*, *DPF-R*, *CgH-L*, and *ACg-WM-R*. Two **(B)** of the ten fiber tissues exhibit a unique intermediate rise in *FW-FA* before completely declining. DPF-L, left dorsal prefrontal white matter; AT-WM-L, left adjacent thalamus white matter; GCC, genu of corpus callosum; EC-R, right external capsule; CTG-R, right central tegmental; AA-WM-R, right adjacent amygdala white matter; MB-WM-L, left midbrain white matter; DPF-R, right dorsal prefrontal white matter; CgH-L, left perihippocampal cingulum; ACg-WM-R, right anterior cingulum white matter.

The interactive effects of cART treatment and time were also found in the FW-corrected diffusion properties of the two fiber tracts (see **Figure 3**, **Table 4**). Those who received cART intervention showed a progressive increase in FW-corrected FA values over time, while those who did not receive it exhibited a decreasing pattern, particularly in the left sagittal striatum and right central tegmental fiber tracts. A *post-hoc* analysis revealed that the most apparent differences in *FW-FA* were exhibited on day 336 of *MRI* scanning, i.e., 296 days after cART intervention (cART-, vs. cART+ at T7, **Supplementary Table S8**). We also identified independent effects of time on FW-FA for several fiber tissues (see **Figure 5**, also see **Supplementary Table S4**). Ten (10) fiber tracts demonstrated a significant reduction in FW-FA values over time, independent of cART status. These fiber tracts include the dorsal prefrontal WM, adjacent thalamus WM, genu of corpus callosum, perihippocampal cingulum, central tegmental, anterior cingulum WM, external capsule, adjacent amygdala WM, and midbrain WM.

### 4.8 Associations of extracellular measures and diffusion properties with the clinical measures

**Figure 6:**
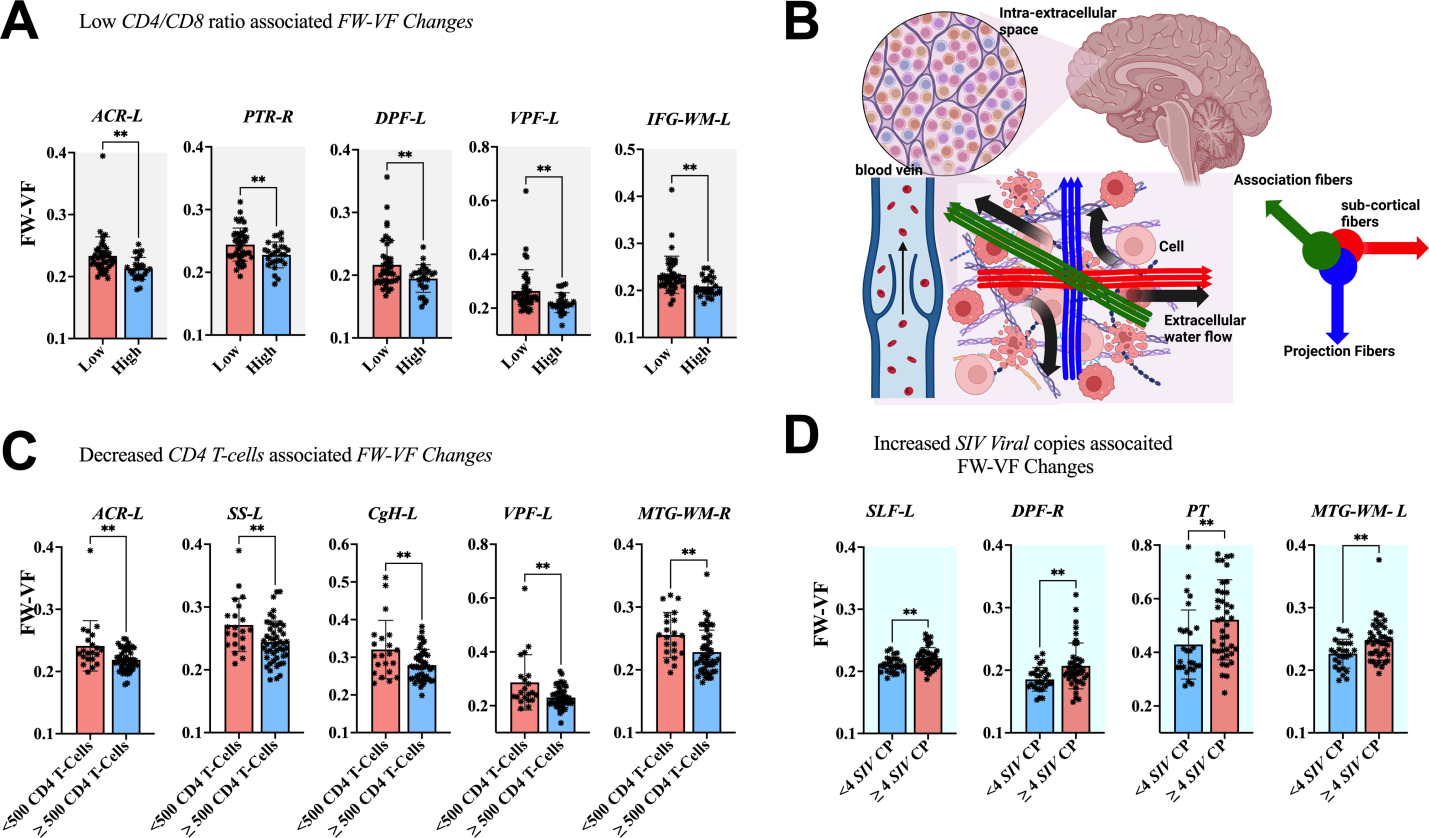
Association between immunological markers and changes in extracellular spaces. The low *CD4/CD8* ratio **(A)**, decreased *CD4* T-cell count **(C)**, and increased number of *SIV* viral copies **(D)** were associated with increased free water volume fraction (*FW-VF*). **(B)** Shows the pathways of extracellular FW in the brain parenchyma and in the proximity of fiber tissues. *ACR-L*, left anterior corona radiata; *PTR-R*, right posterior thalamic radiation; *DPF-L*, left dorsal prefrontal white matter; *VPF-L*, left ventral prefrontal white matter; *IFG-WM-L*, left inferior frontal gyrus white matter; *ACR-L*, left anterior corona radiata; *SS-L*, left sagittal striatum; *CgH-L*, left perihippocampal cingulum; *VPF-L*, left ventral prefrontal white matter; *MTG-WM-R*, right middle temporal gyrus white matter; *SLF-L*, left superior longitudinal fasciculus; *DPF-R*, right dorsal prefrontal white matter; *PT*, pyramidal tracts; *MTG-WM-L*, left middle temporal gyrus white matter. **P < 0.01.

**Figure 7:**
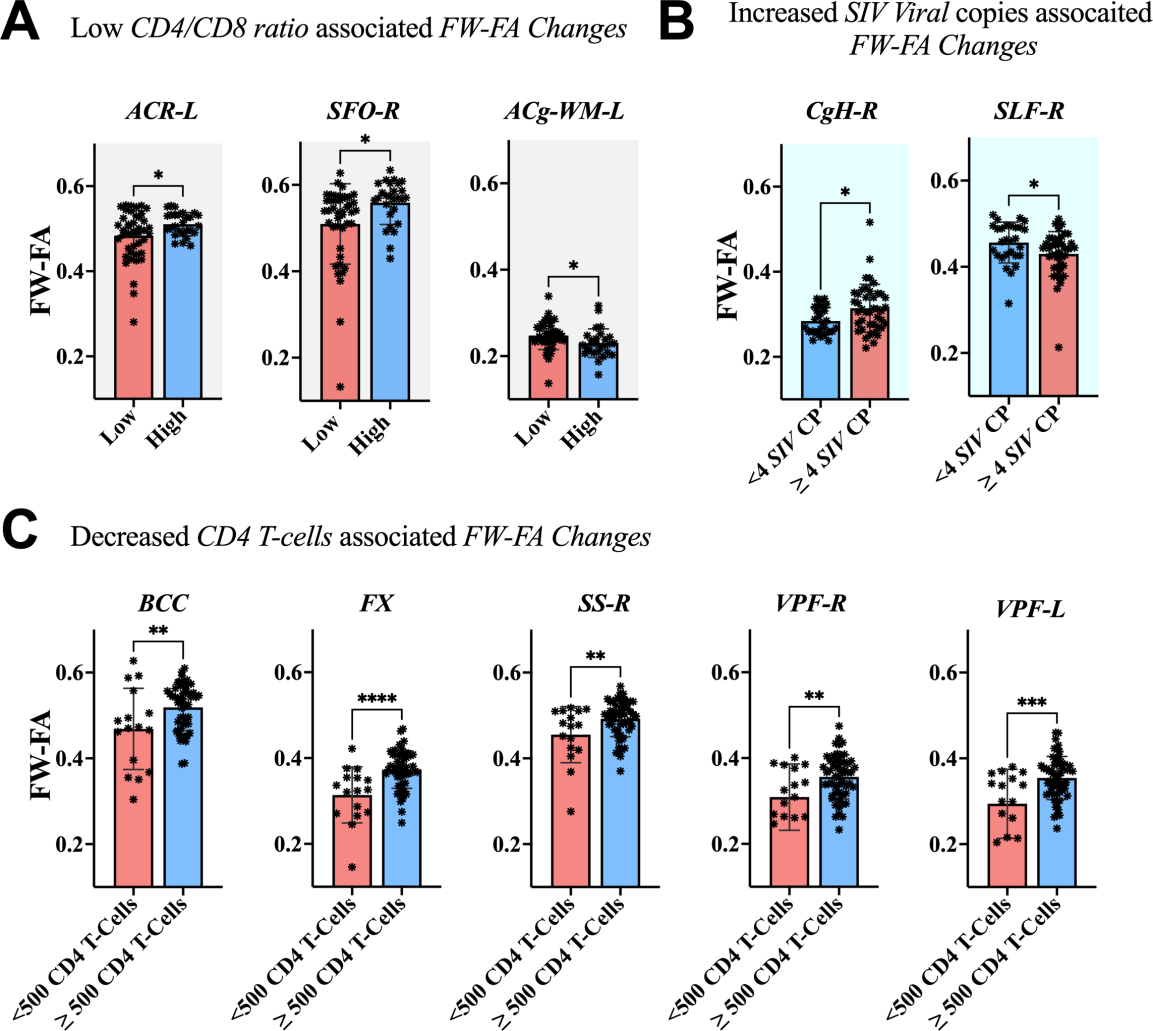
Association between immunological markers and changes in *FW-FA* of fiber tissues. The low *CD4/CD8* ratio **(A)**, decreased *CD4* T-cell count **(C)**, and increased number of *SIV* viral copies **(B)** were associated with decreased *FW-FA* across fiber tissues, with exception of CgH-R and ACg-WM-L presented increased *FW-FA* with increased number of *SIV* viral copies and low *CD4/CD8* ratio, respectively. ACR-L, left anterior corona radiata; SFO-R, right superior fronto-occipital fasciculus; ACg-WM-L, left anterior cingulum white matter; CgH-R, right perihippocampal cingulum; SLF-R, right superior longitudinal fasciculus; BCC, body of corpus callosum; FX, fornix; SS-R, right sagittal striatum; VPF-R, right ventral prefrontal white matter; VPF-L, left ventral prefrontal white matter. *P < 0.05, **P < 0.01, ***P < 0.001, ****P < 0.0001.

**Figure 8:**
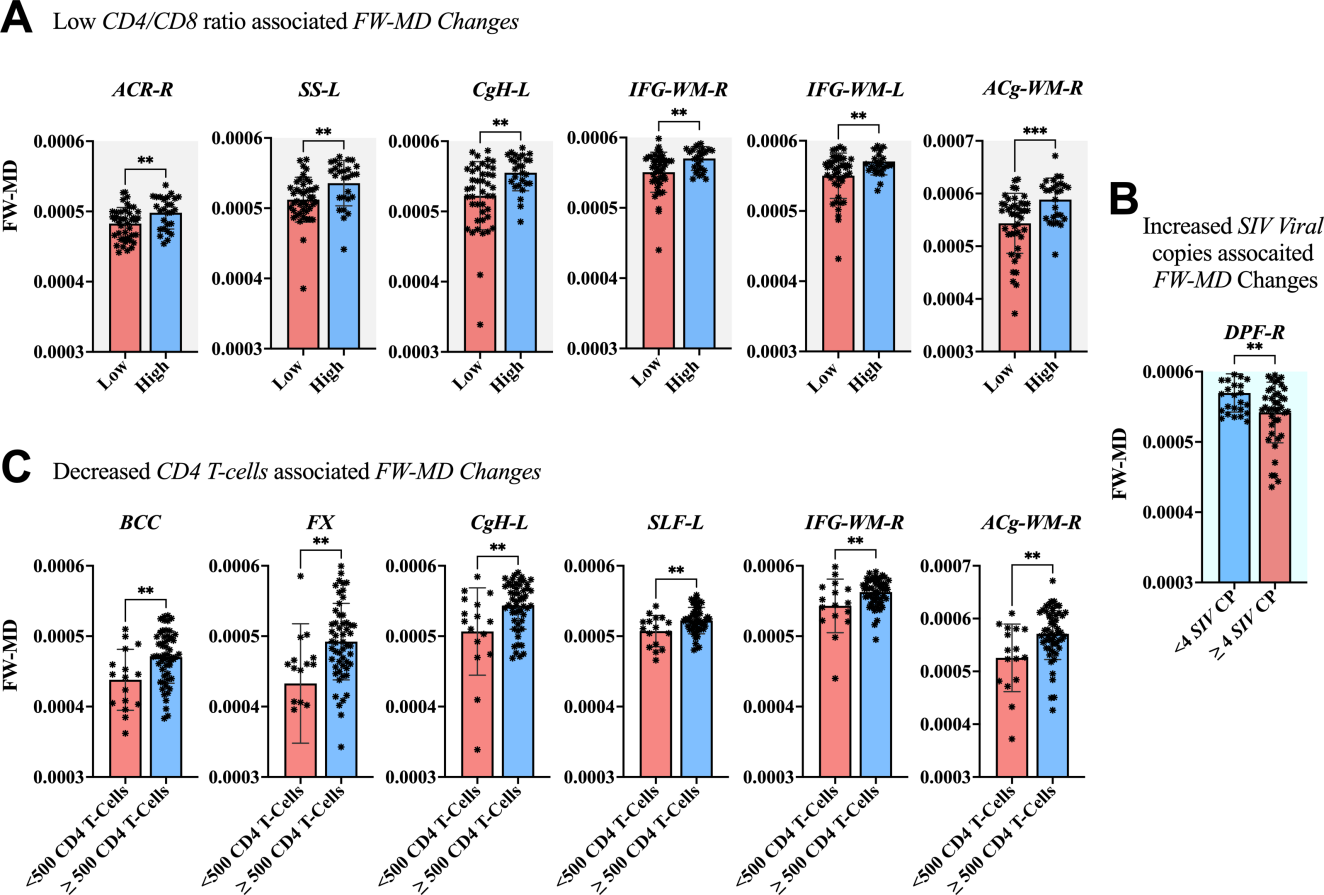
Association between immunological markers and changes in *FW-MD* of fiber tissues. The low *CD4/CD8* ratio **(A)**, decreased *CD4* T-cell count **(C)**, and increased number of *SIV* viral copies **(C)** were associated with decreased *FW-MD* values across fiber tissues. ACR-R, right anterior corona radiata; SS-L, left sagittal striatum; CgH-L, left perihippocampal cingulum; IFG-WM-R, right inferior frontal gyrus white matter; IFG-WM-L, left inferior frontal gyrus white matter; ACg-WM-R, right anterior cingulum white matter; BCC, body of corpus callosum; FX, fornix; CgH-L, right perihippocampal cingulum; SLF-L, left superior longitudinal fasciculus; IFG-WM-R, right inferior frontal gyrus white matter; ACg-WM-R, right anterior cingulum white matter; DPF-R, right dorsal prefrontal white matter. *P < 0.05, **P < 0.01, ***P < 0.001.

The association between clinical measurements (i.e., SIV copies, CD4 T-cells, and CD4/CD8 ratio) and brain markers is shown in **Figures 6**–**8**. A progressive increase in viral load (SIV copies per mL) and a continuous decline in the immune system (indicated by lower CD4 T-cells and CD4/CD8 ratio) occurred in parallel with changes in the extracellular spaces and diffusion properties of brain fiber tissues. In fact, a low *CD4/CD8* ratio was associated with increased extracellular *FW-VF* across fiber tissues of the ACR-L, PTR-R, DPF-L, VPF-L, and IFG-L (**Figure 6A**). Similarly, decreased CD4 T-cell counts were strongly associated with increased *FW-VF* across the ACR-L, SS-L, CgH-L, VPF-L, and MTG-WM-R (**Figure 6C**). Conversely, an increased number of SIV copies, reflecting disease progression, was highly associated with increased FW-VF in the fiber tissues, particularly the SLF-L, DPF-R, PT, and MTG-WM-L (**Figure 6D**)(Also see **Supplementary Table S5** for statistics). For diffusion properties, we found significant associations between compromised immunity, increased SIV copies, and the diffusion properties of the fiber tissues (**Figure 7**). In particular, a low CD4/CD8 ratio was associated with reduced FW-FA values; this was more apparent in the fiber tissues of ACR-L, SFO-R, and ACg-WM-L (**Figure 7A**). Decreased CD4 T-cells were also associated with reduced FW-FA values across multiple fiber tissues, especially the BCC, FX, SS-R, VPF-R, and VPF-L (**Figure 7C**). Conversely, increased *SIV* viral copies per ml were associated with reduced *FW*-*FA* values in the CgH-R and increased *FW*-*FA* values in the SLF-R (**Figure 7B**) (Also see **Supplementary Table S6** for statistics of these results). On the other hand, the analysis of associations with FW-MD revealed that a low *CD4/CD8* ratio was independently associated with reduced *FW*-*MD* values across the ACR-R, SS-L, CgH-L, IFG-WM-R, IFG-WM-L, and ACg-WM-R (**Figure 8A**). Additionally, decreased CD4 T-cells were associated with reduced *FW*-*MD* values for fiber tissues of BCC, FX, CgH-L, SLF-L, IFG-WM-R, and ACg-WM-R (**Figure 8C**), whereas increased viral copies as the disease advances was associated with reduced *FW*-*MD* values in the DPF-R fiber tissues (**Figure 8B**) (Also see **Supplementary Table S7** for statistics of these results). Further analysis using correlation techniques to support the findings of this section is summarized in **Supplementary Table S9**. And the most interesting results were in **Supplementary Table S10**, where the rate of changes in CD4 T-cells over time was significantly correlated with the rate of changes in *FW-VF, FV-FA*, and *FW-MD*.

## 5 Discussion

Here, to gather evidence from animal models to answer the seven key questions of interest, we injected rhesus monkeys with Simian Immunodeficiency Virus (*SIV*). The first question was to determine the SIV entry into the CNS compartment of Chinese-origin rhesus monkeys as early as 7 days post-inoculation. Second, we focused on investigating the time at which the effects of *SIV* begin to manifest in the extracellular environment and observed whether these effects occur in parallel with immune deterioration. Third, we investigated the spectrum of injury to the extracellular environment when the rhesus monkeys are not initiated with suppressive antiretroviral therapy over an extended period of time. Forth, we examined whether or not our combination of the emtricitabine (*EMTBL/FTC*) + dotutegravir (*DTG*) + tenofovir disoproxil fumarate (*TDF*) could repair the extracellular damage induced by *SIV*. Fifth, we focused on investigating whether our regimen exacerbates, to a certain extent, the detrimental effects of *SIV* on some brain regions. Sixth, we examined whether the improvement in the immune system, if it exists after taking the regimen, is parallel to the improvement in the intra- and extracellular milieu of the brain. Lastly, we gathered evidence to validate whether inflammatory processes are likely or partly at the core of changes in free water volume.

In line with the first question, our data demonstrate the immunity level does not drop immediately after the infection but may manifest deficiency within the first 7 days after infection. Within this period, the number of *SIV* copies per ml increases rapidly from 0 to 6.89 ± 0.44 in plasma, or to 6.92 ± 0.40 in CSF. This rise of viral number signals the constant attacks of the immune system by *SIV*. The *CD4/CD8* ratio begins to drop by 17.5% from 2.00± 0.77 at baseline to 1.65 ± 0.93 on day 7 following the infection. A continuous attack of the immune system causes a significant fall in the number CD4 T-cells below 1000 cells/μl and a further decline of *CD4/CD8* ratio below ~1 within the first 14 days of infection. This marked effects on the immune system observed within the first 14 days after infection continue getting worse if no intervention is made as early as day 35 to 40 after infection. At this rate, the number of *SIV* copies/ml in plasma rises from 6.89 to 7.17 copies/ml. An early deficiency in the immune system seen in the first 14 days is accompanied by a 6% weight loss in rhesus monkeys; however, the animals regain their weight by day 28 and continue to show a progressive increase over time with a consistent, progressive diet.

Regarding to whether the *SIV* effects in the extracellular spaces and diffusion properties occur in parallel with immune deterioration, we found that increased *SIV* copies and impaired immune system (declined cd4 T-cell counts and reduced *CD4/CD8* ratio) in rhesus monkeys occur in parallel with *SIV*-induced brain alterations in extracellular environment and diffusion properties of major fiber tissues. Both increased viral copies of *SIV* and impaired immune system are directly associated with increased changes in extracellular spaces (increased *FW-VF*) and reduced white matter integrity (reduced *FW*-*FA* values). Our findings of early deficiency in the immune system post-infection, which correspond to changes within the brain, are consistent with those of Gopalakrishnan et al., who detected increased IL-6 expression and other proinflammatory responses in the brain as early as 7 days of *SIV* infection, possibly indicating that the early *SIV* infection in the brain is characterized by significant macrophage infiltration and astrocyte activation (24). These activities are likely to contribute to increased levels of extracellular vesicles and elevate the levels of miR-21 in extracellular vesicles, leading to necroptosis via TLR7 signaling (25).

In addition to these previous studies, our experimental findings provide a clearer evidence that it takes about 28 days or less for the *SIV* effects on the brains of the Chinese-origin rhesus monkeys rhesus to surface and manifest, at least to a level detectable by MRI imaging. After *SIV* entry to the brain, the extracellular *FW* volumes of various fiber tissues are likely to exhibit disturbance within the period of 28 days. The changes in the extracellular spaces is probably the aftermaths of viral impact to the immune system (weakened immune system and brain defense system). This includes weakened brain-blood barrier (BBB), thereby allowing the *SIV* filtration to the parenchymal environment of the brain (26, 27). The entry of the *SIV* into the brain parenchyma may induce significant changes in both the extracellular and intracellular spaces. Our data show that we can capture these changes using MRI within the first 28 days of infection. Largely, *SIV* infection appear to cause excess free water content in the extracellular spaces, and this excess free water is largely seen within the fiber tissues of the internal and external capsules, corona radiata, thalamic radiation, longitudinal fasciculus, uncinate fasciculus and the prefrontal WM. The other possible changes that manifest within this period following *SIV* infection include alterations in the *FW*-corrected diffusion properties (*FW*-*FA* and *FW*-*MD*) of the fiber tissues. Based on our experiment, the *FW*-*FA* values decrease progressively after infection in a number of fiber tissues and such a decrease was more severe in the left central tegmental, at least within this period of 28 days after infection. The identified changes in *FW*-*MD* within period are prevalent across several fiber tissues, with two major fibers — the cerebellar peduncle (right and left inferior, and left superior) and the internal capsule (retrolenticular limb) indicating significant increases in *FW*-*MD* values.

Regarding the extent of injury to which the extracellular environment could sustain when the rhesus monkeys are left untreated over extended period of time, our data reveal that *SIV* would likely continue to disrupt the extracellular environment over time, thereby increasing the fractional volumes of the *FW* in extracellular spaces of the left sagittal striatum and the right tegmental fiber tract. As the *CD4/CD8* ratio and CD4 T-cell number keep dropping, and *SIV* viral copies keep increasing, the extracellular *FW-VF* surrounding several fiber tissues are likely to be affected, including the left anterior corona radiata, right posterior thalamic radiation, left dorsal prefrontal WM, left ventral prefrontal WM, left inferior frontal gyrus WM, left perihippocampal cingulum, right middle temporal gyrus WM, left superior longitudinal fasciculus, and pyramidal tracts. As the infection progresses, some of these fiber tissues and others may start to display changes in their *FW*-*FA* values, suggesting a severe threat to their microstructural integrity. The fiber tissues likely to undergo such modifications include the left anterior corona radiata, right superior fronto-occipital fasciculus, left anterior cingulum WM, right perihippocampal cingulum, right superior longitudinal fasciculus, body of the corpus callosum, fornix, right sagittal striatum, and left/right ventral prefrontal WM. On the other hand, the *FW*-corrected *MD* values of these fiber tissues indicate changes. The more notable changes in *MD* values start to appear in the body of the corpus callosum, fornix, left perihippocampal cingulum, left superior longitudinal fasciculus, left/right inferior frontal white matter, right anterior cingulum white matter, right anterior corona radiata, and left sagittal striatum. Meanwhile our findings admit that the rhesus monkey may exhibit different spectrum of injury on the extracellular environment and fiber tissues, which may depend on the variations in the trajectory of disease progression, partially dependent on the levels of immunity deterioration and nadir *CD4* T-cell counts, among other factors.

Whether the spread of *SIV* injury in the extracellular spaces or across fiber tissues follows specific physical pathways or is promoted by certain physical organization of tissues in relation to their proximity to the vasculature or CSF pathways requires further investigation. However, what we know so far is that the central tegmental and sagittal striatum fiber tissues, whose surrounding extracellular *FW-VF* has been attenuated, have consistently been linked to HIV pathology (28–30). For example, a study of a 52-year-old male with HIV and a history of hypertension and cocaine abuse detected abnormal WM lesions on the bilateral central tegmental fiber tract and a subacute infarct of the posterior limb of the right internal capsule (28). The central tegmental fiber tissue and other white matter tracts such as longitudinal fascicules and anterior commissure of patients with AIDS have been identified with a large and localized accumulation of dense cytoplasm and myelin balloons—the vacuoles in myelin sheaths after HIV infection (31). Such pathogenic features in WM tracts are accommodated in the spaces between myelin lamellae, or arise from the space between axon surface and inner most myelin wrap (32), indicating focal spongy myelin damage or vacuolar myelin damage, caused by HIV-induced inflammation and neurotoxins (31). Studies pointed out that HIV injury also appear in sagittal striatum fiber tissues as alterations in *FA*, *MD*, or AD values, reflecting axon damage or demyelination (29, 30, 33), or as WMH lesions (34). Therefore, our findings, which show an excessive increase in extracellular *FW-VF* and alterations in *FW*-*FA* and *FW*-*MD* values in various fiber tissues, including the sagittal striatum and central tegmental, in *SIV*-infected rhesus monkeys untreated with cART, may provide additional MRI-based evidence of earlier reports of brain injury after infection. The findings also offer further evidence of how the injury progresses across other fibers when the rhesus monkeys are left untreated.

Our data indicate that the FTC+*TDF*+*DTG* regimen not only enhances the immune system and suppresses *SIV* replication but also promotes the repair of extracellular damage caused by *SIV*. Within the first 44 days of progressive cART the number of *SIV* copies in cART-treated rhesus monkeys was significantly reduced by 75.09%, with 60%(3 of 5), 80%(4 of 5), and 100% (5 of 5) of rhesus macaques achieving a total viral suppression by day 44, day 128, and day 212, respectively. The immunity level was maintained to nearly 1.5 (*CD4/CD8* ratio) from day 16 through day 212 following cART initiation, and significantly restored to 1.9 on day 310 of suppressive cART. Our observations revealed that these rhesus monkeys who responded positively to suppressive drugs (i.e., attaining viral suppression and improving immune system), demonstrated an improved extracellular environment as quantified by *FW-VF* as well as improved white matter integrity as quantified by *FW*-*FA*. These findings cement that enhancements in the immune system post regimen initiation align with positive alterations in both the intra- and extracellular environments of the brain. This underscores the critical importance of early commencement of cART to manage viral replication or limit viral reservoir effectively (3, 35), thereby playing a crucial role in averting further harm to the brain. Our data do not provide sufficient evidence to indicate an increase in detrimental effects of suppressive antiretroviral therapy on the damage caused by *SIV*. We observed improved *FA* and reduced *FW-VF* over time in most fiber tissues following the initiation of suppressive antiretroviral therapy. However, there are main effects of time on rhesus monkeys following the infection with three different patterns of trends of across fibers. The first trend indicated a decrease in *FW-VF* as time progressed, whereas the other two trends displayed a consistent increase in *FW-VF* over time, regardless of the treatment administered to the monkeys.

Our experimental findings likely suggest the occurrence of modifications in the extracellular milieu post-infection in inflammatory diseases, underscoring the impact of inflammatory processes on modulating extracellular spaces and functions. This conclusively addresses the early conjectures associating inflammatory disorders such as Parkinson’s disease with changes in extracellular volume (6, 16). The presence of inflammatory cytokines and inducible nitric oxide synthase (iNOS) in the brains of *SIV*-infected monkeys and increased levels of nitric oxide (NO) in the cerebrospinal fluid (CSF) (36) underscores the ongoing inflammation and immune activation, which may underlie changes in extracellular environment, including *FW-VF*. Our research provides further evidence for the possibility that elevated extracellular *FW-VF* serves as a reliable biomarker for neuroinflammation in neurodegenerative conditions, aligning with early studies (37), and we further cement that an impairment in glymphatic function by neuroinflammation can further exacerbate the abnormal accumulation of cerebrospinal fluid (CSF) and extracellular fluid in the brain, progressively leading to alterations in the white matter integrity, cellular signaling, and homeostatic balance of the brain tissues (38).

### 5.1 Limitations

There are several limitations worth-mentioning in this study. First, while we generally agree that HIV or SIV can be considered neuroinflammatory diseases due to HIV or SIV-triggered activation of microglia, NLRP3 inflammasome, and caspase-1, as well as the release of IL-1β (39–41), the absence of a study-specific histopathological assessment may limit our conclusions. Second, although we applied several exclusion criteria to our rhesus monkeys to screen out those with conditions such as herpes B virus, STLV-I, and SRV, which may affect the brain, further studies are needed to account for other potential factors, such as early inflammation status, which is likely to affect the DTI-derived metrics. The current study design did not include a control cohort for comparison, which could have strengthened our findings. Thus, a control cohort is warranted to broaden the scope of our findings.

## 6 Conclusion

Our findings show that the immunity level is compromised as early as 7 days after *SIV* infection, with a rapid increase in *SIV* copies per mL and a significant drop in the *CD4/CD8* ratio within the first 14 days of infection. The compromise in the immune system occurs in parallel with alterations in the extracellular environment and diffusion properties of fiber tissues, possibly suggesting that *SIV* enters the brain parenchyma in the very early days of infection via a weakened brain defense system. Our experimental findings provide direct and longitudinal evidence of the benefits of early initiation of FTC+*TDF*+*DTG* in enhancing the immune system, suppressing *SIV* replication, and repairing and curbing further damage to the intra- and extracellular environment. And such benefits may be more apparent as early as the first 44 days of cART initiation. Lastly, our findings provide the first SIVmac239-based evidence that extracellular *FW-VF* may be a reliable biomarker of increased inflammatory processes in neurological disorders, and as thus this information may be valuable to development of therapeutic strategies.

## Data Availability

The original contributions presented in the study are included in the article/**Supplementary Material**. Further inquiries can be directed to the corresponding author.
